# S12-2: Perceptions of the #Wethe15 campaign to promote rights of physical activity among people with disabilities

**DOI:** 10.1093/eurpub/ckae114.252

**Published:** 2024-09-26

**Authors:** Kwok Ng, Kwok Ng, Damian Haslett, Jessica Noske-Turner, Emaa Pullen, David Legg

**Affiliations:** University of Limerick, Ireland; University of Turku, Finland; Lithuanian Sports University, Lithuania; University of Limerick, Ireland; University of Turku, Finland; Lithuanian Sports University, Lithuania; Loughborough University London, United Kingdom; Loughborough University London, United Kingdom; Loughborough University London, United Kingdom; Mount Royal University, Canada

## Abstract

**Purpose:**

Since 1948, the Paralympic Games are a platform that gives opportunities to persons with disabilities for competitions akin to the Olympic games. This may be seen as fulfilment of the United Nations Convention on the Rights of Persons with Disabilities . Article 30, where people with disabilities have the to participate in cultural life, recreation, leisure and sport on an equal basis with others. It is less known, how the disability community see the acceptability of these games. A campaign to raise awareness is through the #Wethe15 campaign. This study aimed to investigate the initial perceptions of the #Wethe15 campaign after the 2021 Paralympic Games.

**Methods:**

Interviews and focus groups with representatives of disability rights groups from Canada, Finland, Malawi, Peru and the United Kingdom were carried out to investigate perceptions about the #Wethe15 campaign. The main questions were related to how the disability community could contribute or benefit from the campaign. Data were then translated, then transcribed in English and analysed through thematic analyses mapped against the concepts of availability, accessibility, and acceptability.

**Results:**

In late 2021, 29 representatives (20 with lived experience of disability) took part in five focus groups and interviews. The campaign was deemed as acceptable due to the positive sentiment about the campaign to promote disability rights and sport in society. Yet, as #Wethe15 does not translate well into some non-English languages, more instructions of how to make it culturally appropriate was needed to broaden access to the main message of the campaign.

**Conclusions:**

The campaign brought awareness of disabilities in society, yet the rights of physical activity to people with disabilities was less obvious, particularly regarding availability and accessibility. Partners of the movement need to find ways to fund mechanisms to promote more quality, accessibility and availability, while recognising the health benefits from participation in physical activity.

**Funding:**

The study was commissioned by the IPC. The IPC had no influence the recruitment of the participants or interpretation of the results.

